# A DEGREE IN SOCIAL WORK INCREASES FAMILY ENGAGEMENT WITH THE SOCIAL SERVICES DEPARTMENT

**DOI:** 10.1093/geroni/igac059.1567

**Published:** 2022-12-20

**Authors:** Amy Roberts, Nancy Kusmaul, Amber Alaniz, Mercedes Bern-Klug

**Affiliations:** Miami University, Oxford, Ohio, United States; University of Maryland Baltimore County, Baltimore, Maryland, United States; University of Iowa, Iowa City, Iowa, United States; University of Iowa, IOWA CITY, Iowa, United States

## Abstract

This mixed-methods presentation will examine the role of social services directors in supporting the family members of nursing home residents. A logistic, hierarchical regression (n= 924) found a positive association between the social services director having a degree in social work and strong agreement with "family members regularly engage the expertise of the social services department." A qualitative analysis of written-in comments to the question, “What do you like about your job?” identified 234 comments that described directors’ view of the importance of working with and supporting families. A content analysis revealed that two types of support are provided to families, instrumental and emotional. Instrumental support includes connection to resources for adjustment, financial needs, and discharge planning. Emotional support includes activities such as helping people through transitions, and coping with grief and loss. Appropriately trained social services directors can offer different types of support and be valued resources for families.

